# Myocarditis and Myocardial Disease—III

**Published:** 1908-08-22

**Authors:** 


### Medicine.

### MYOCARDITIS AND MYOCARDIAL DISEASE—III.

IV. Acute General Interstitial Myocarditis.

Acute general interstitial myocarditis is a very rare disease, although it is the lesion which many people have in their minds when they speak of "acute myocarditis." In cases of acute pericarditis the layer of muscle tissue immediately under the pericardium is nearly always pallid for a variable depth all round the heart. There is extensive exudation of lymph and leucocytes, and small round cells in and upon the visceral pericardium. It might be thought that there would almost certainly be a similar exudation of small round cells between the fibres of at least the superficial layers of the myocardium—in other words, an acute interstitial myocarditis. This, however, is seldom the case. The small round-celled exudation is almost or quite confined to the surface, the pallor of the underlying muscle being due to cloudy swelling and segmentary degeneration.

In a very small number of cases, however, both of acute rheumatic affection of the myocardium without pericarditis, and of a similar affection with pericarditis, and in a few cases of suppurative pericarditis, there is an actual small round-celled infiltration of the connective tissue of the heart, associated with cloudy swelling of the parenchyma. It is so rare, however, that when one diagnoses "acute affection of the myocardium" at the bedside, the mental picture that one should have is that of acute cloudy swelling of the parenchyma with little or no interstitial inflammation, for the chances of the latter being present in anything like a marked degree are very small.

V. Acute Focal Interstitial Inflammation.

Acute focal interstitial inflammation is in reality a form of pyæmia. It must be remembered that pyæmic lesions are not necessarily abscesses, though there is no sharp line of demarcation between a focus consisting of bacterial colonies and exuded small round cells which have not broken down into pus, and a similar focus in which pus has arisen. The focal interstitial inflammation occurs in connection with pyæmic infection, and is caused by micro-organisms which either attack the myocardium directly from the endocardium or are brought to it by the blood-channels. So far as is known, the bacteria that are brought to the heart muscle in the blood are the same as those found in the fungating endocarditis which is usually present at the same time. They may be derived from obvious sources of absorption, such as suppurating wounds, though in some instances they gain access to the body without leaving any recognisable traces at their point of entrance. When in some such manner multitudes of bacteria reach the heart muscle the affected person may speedily succumb, and at the autopsy the cardiac wall is seen to be beset with numerous small opaque greyish-yellow spots which represent bacterial colonies. Within these the muscle is degenerate or completely destroyed, and there is a considerable degree of inflammatory small round-celled infiltration in the immediate neighbourhood.

Small deposits of pus may be reabsorbed, no doubt, and leave small fibrous scars or calcareous specks. Death occurs in most cases, however, and if it is a little postponed, yellowish-white accumulations of actual pus may be found in the affected areas.

VI. Fatty Over-growth or Superposition.

In the condition of fatty over-growth of the heart, the cardiac surface is overlaid by a large quantity of fat, so that the muscular fibres may be entirely concealed from view. The fat, which is simply an over-growth of adipose tissue beneath the visceral pericardium, does not necessarily interfere with the heart's action at all; it is only part of a general excess of fat or obesity. In some cases, however, the muscle fibres immediately beneath the mass of fat undergo atrophy, so that the actual bulk of the contractile portion of the heart is diminished, and yet has to do the work required by an enormous body. Symptoms of cardiac failure will result sooner or later. Moreover, fatty over-growth is very apt to be associated with fatty infiltration, with all the ill-effects of the latter.

VII. Fatty Infiltration.

Fatty infiltration of the heart is a condition in which there is more or less extensive deposition of fat in the interstices between the muscle fibres. There may or may not be an excess of sub-pericardial fat upon the surface of the heart at the same time. The microscopic section of the heart wall shows that, instead of there being a sharp and uniform line of demarcation between the surface-fat on the one hand and the muscle beneath it on the other, this line has become irregular owing to extension of the fat between the layers of muscle, especially along the lines of the arterioles. This extension always occurs from without inwards, and never from the endocardium outwards; even in the fattest hearts there is no adipose layer between the endocardium and the myocardium.

Separation of the bands of muscle fibres by deposits of inert fat should lead to defective contractile force. The condition of affairs is, however, worse than this; for not only are the bands of muscle fibres mechanically separated from one another, but there is also considerable atrophy of those fibres which are adjacent to the fatty deposits; the cardiac force is therefore still further diminished.

There are probably two forms of fatty infiltration of the heart; the one in which the fat is rapidly stored amongst normal fibres, interfering with their functions and nutrition secondarily; the other in which the fatty change merely, as it were, fills up the interstices left by an atrophy of muscle tissue that has been taking place before the fat came there. Of these varieties the first is by far the commoner. It is met with in persons of inactive and often indolent and self-indulgent lives, in men at middle age, in women towards the climacteric period or soon after it. It is people who have good appetites and good primary digestion with faulty eliminative power that are especially liable to the disease. Indulgence in alcohol, especially malt liquors and sweet wines, certainly favours its occurrence. No organic disease of any kind is necessary as a forerunner of the trouble, which may arise solely from an excess of alimentary supply over demand, however brought about.

There may be few symptoms, and the first indication of the poor state of the heart may be the behaviour of the pulse during an attack of bronchitis or pneumonia, or during and after a surgical operation. The patients are particularly bad subjects for taking anæsthetics.

If symptoms of heart trouble arise, apart from intercurrent affection, they chiefly take the form of undue shortness of breath on any unusual exertion, a tendency to perspire easily, and a venous tinge in the general complexion. Later, all the signs of chronic heart failure may be observed.

The Nauheim treatment by graduated exercises and strict dieting produces better results in this form of heart disease than in almost any other.

